# Medial Plantar Proper Digital Nerve Thickening – a Sonographic Finding in Volunteers Without Joplin’s Neuroma

**DOI:** 10.1055/a-2675-5871

**Published:** 2026-01-14

**Authors:** Veronika Vetchy, Tobias Rossmann, Paata Pruidze, Wolfgang Grisold, Wolfgang Weninger, Stefan Meng

**Affiliations:** 1 Biomedical Imaging and Image-guided TherapyMedical University of ViennaViennaViennaAustria; 2 Department of Neurosurgery, Neuromed Campus 31197 Kepler University HospitalLinzUpper AustriaAustria; 3 Center for Anatomy and Cell BiologyMedical University of ViennaViennaAustria; 4 Neurology Consultancy Unit, Division of Anatomy 27271 Medical University of ViennaViennaViennaAustria; 5 Radiology 31315 Hanusch HospitalWienAustria

**Keywords:** medial plantar nerve, nerve thickening, asymptomatic, medial proper plantar digital nerve, high-resolution ultrasound

## Abstract

**Purpose:**

Thickening of the medial plantar proper digital nerve (MPPDN) along the medial aspect of the big toe is frequently observed incidentally during high-resolution ultrasound examinations in asymptomatic individuals. However, the clinical significance of these findings remains unclear, as no study has definitively linked them to symptoms. Although the localization and visualization of the medial plantar nerve using ultrasound have been documented, its cross-sectional area has not yet been utilized to assess thickening at this specific site. The study aimed to assess the frequency and specific locations of MPPDN thickening in asymptomatic individuals and to identify associated characteristics.

**Materials & Methods:**

In this prospective study, high-resolution ultrasound examination of the MPPDN was performed on asymptomatic volunteers. The cross-sectional areas and their changes along the course of the nerve are evaluated descriptively. Potential contributing demographic factors in participants are captured.

**Results:**

56 asymptomatic volunteers, 30 women and 26 men (median age: 28, range: 20–69) participated in this study. 22 individuals (39.3%) showed thickening of the MPPDN at the interphalangeal joint either in one or both feet. More participants above the median population age of 28 showed thickening of the MPPDN (15 [57.7%] versus 7 [23.3%] p = 0.009).

**Conclusion:**

Thickening of the MPPDN is frequently observed in asymptomatic individuals, with the likelihood of these findings increasing with age. The pathological significance should however not be overestimated and should be assessed in a clinical context.

## Zusammenfassung

**Ziel:**

Eine Verdickung des medialen plantaren Digitalnervs (MPPDN) entlang der Großzehe wird bei beschwerdefreien Personen häufig zufällig bei Ultraschalluntersuchungen beobachtet. Die klinische Bedeutung ist bisher unklar, da keine Studie diese Veränderungen eindeutig mit Symptomen verknüpft hat. Ziel war die Erfassung der Häufigkeit und Lokalisation der MPPDN-Verdickungen bei asymptomatischen Individuen und die Identifikation assoziierter Patientencharakteristika.

**Material und Methoden:**

In dieser prospektiven Studie wurde eine hochauflösende Ultraschalluntersuchung des Nervs bei asymptomatischen Freiwilligen durchgeführt. Die Querschnittsflächen und deren Veränderungen entlang des Nervenverlaufs wurden deskriptiv ausgewertet und mögliche demografische Faktoren erfasst.

**Ergebnisse:**

Sechsundfünfzig asymptomatische Freiwillige, 30 Frauen und 26 Männer (medianes Alter 28, Bandbreite 20–69), nahmen teil. Bei 22 Personen (39,3%) zeigte sich eine Verdickung im Bereich des ersten Interphalangealgelenks in einem oder beiden Füßen. Über dem medianen Alter von 28 Jahren wiesen mehr Patienten eine MPPDN-Verdickung auf (15 [57,7] versus 7 [23,3] p = 0.009).

**Schlussfolgerungen:**

Die Verdickung des medialen plantaren Digitalnervs wird häufig bei asymptomatischen Personen beobachtet, wobei die Wahrscheinlichkeit mit steigendem Alter zunimmt. Die pathologische Bedeutung sollte jedoch nicht überbewertet und im klinischen Kontext beurteilt werden.

## Introduction

Thickening of the medial plantar proper digital nerve (MPPDN) ( Fig. 1 ) is often referred to as Joplin’s neuroma. This nerve thickening is described as an entrapment or impingement of the medial plantar digital nerve, usually at the level of the head of the first metatarsal bone 1 2 . Patients typically experience paresthesia (tingling or numbness) and pain on the medial side of the hallux 1 2 . The diagnosis of Joplin`s neuroma is made by evaluating clinical symptoms and patient history, like previous bunion surgery or trauma 1 3 . Studies have demonstrated that ultrasound can effectively assess these nerves, their anatomical variations, and pathological changes 4 5 6 .

**Fig. 1:**
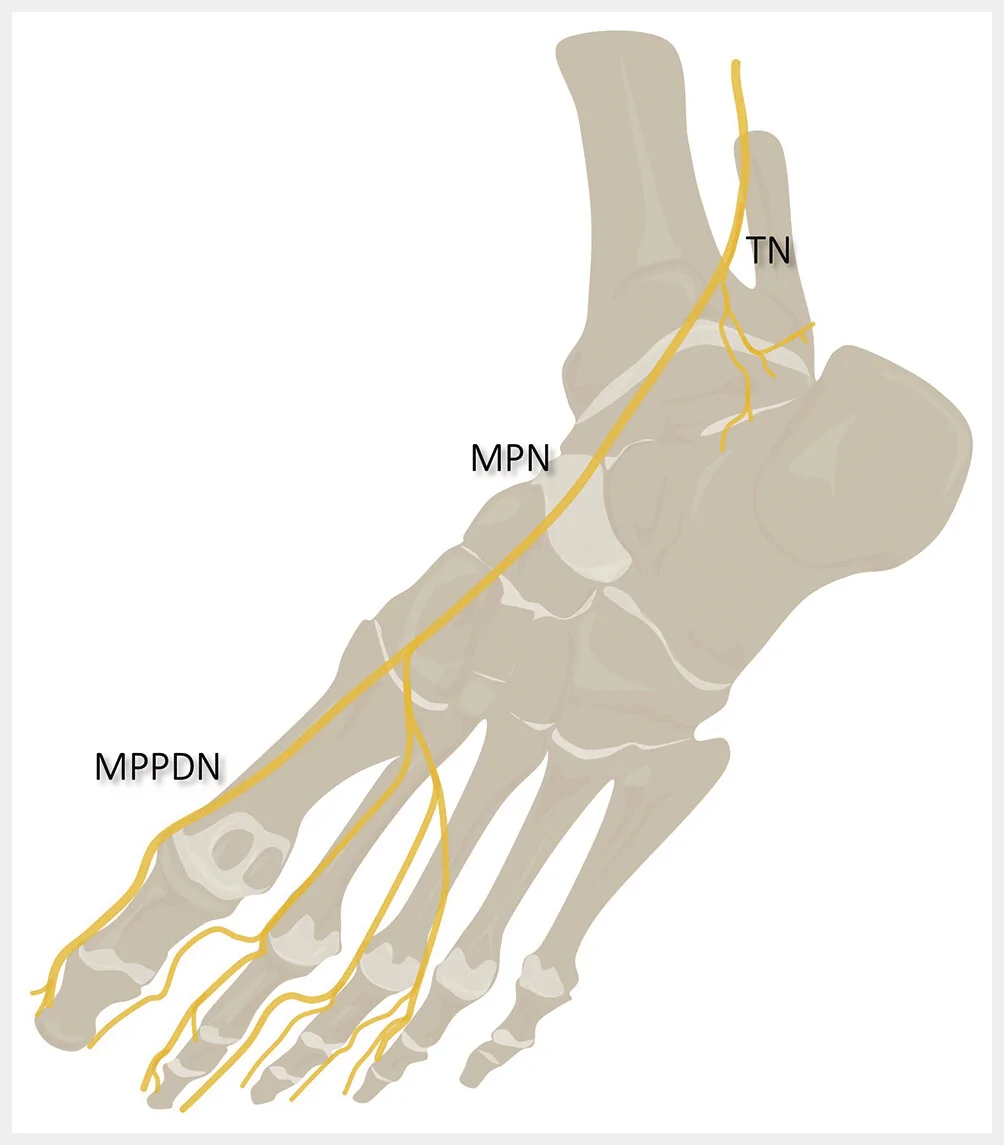
The tibial nerve (TN) enters the tarsal tunnel and divides into the medial (MPN) and lateral plantar nerves. The medial proper plantar digital (MPPDN) nerve branches off from the medial plantar nerve and runs along the medial side of the big toe.

Ultrasound is a reliable tool to visualize and evaluate nerves, highlighting normal anatomy and showing even small changes in nerve diameter and fascicle architecture. Nerve thickening caused by compression, traction, or similar factors can be measured by an increase in the nerve’s cross-sectional area (CSA) 7 8 9 10 11 .

These nerve changes are frequently observed in asymptomatic individuals during clinical practice. However, their clinical relevance and potential implications for diagnosing or predicting an underlying pathology remain uncertain.

This study set out to determine the frequency and location of MPPDN thickening in asymptomatic volunteers in relation to adjacent anatomical landmarks.

## Methods

### Study procedure

This prospective study was conducted in accordance with the Declaration of Helsinki and was approved by the local ethics committee. All participants gave written informed consent to participate in the study. A total of 56 healthy volunteers were recruited through a public announcement at the local institution over the time frame of one year through convenient volunteer sampling. Any adult aged 18 years or older was invited to participate. We excluded all individuals with a history of comorbidities like diabetes, polyneuropathies, previous surgeries, or injuries at the region of interest. Single foot participation was accepted. All participants were examined by one of the radiologists instructed by a neurologist for regular palpable arterial pulses, skin defects, foot symmetry, dys- or hypoesthesia, and weakness of the short foot muscles. Participants were labeled “asymptomatic” if the clinical examination of the feet showed no abnormalities.

### Ultrasound examination

All ultrasound examinations were performed by radiologists experienced in MSK ultrasound together in consensus using a standard clinical ultrasound system with a 22 MHz high-resolution transducer (Aplio i800 with i22LH8, Canon Medical Systems Europe B.V.). The MPPDN was examined from the mid-foot to the tip of the big toe. Nerve thickening was defined as an increase in cross-sectional area (CSA) easily notable on ultrasound examination. No specific cut-off value was defined. Nerve thickening, nerve morphology, and the location in relation to the adjacent bony structures were noted. We measured the CSA and length of all instances of nerve thickening ( Fig. 2 ).

**Fig. 2:**
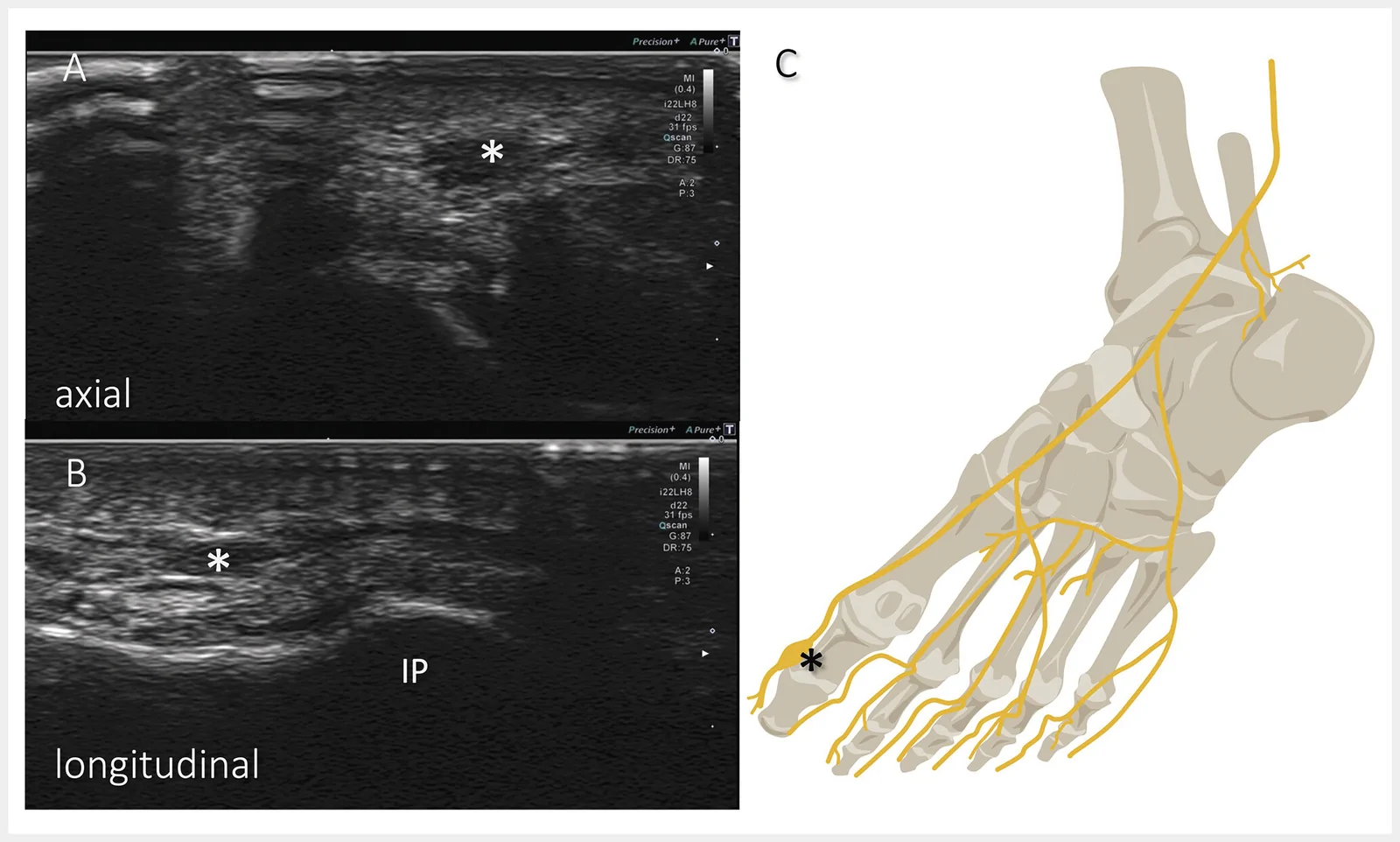
Ultrasound images of the thickened medial proper plantar digital nerve in ( **A** ) axial plane, ( **B** ) longitudinal plane, and ( **C** ) a corresponding anatomical schematic diagram. The asterisk depicts a thickened nerve segment proximal to the interphalangeal joint (IP) of the big toe.

### Statistical analysis

Continuous variables were described as means and standard deviations if they were normally distributed, or otherwise, as medians and ranges. Categorical variables were described using frequency and percentages. Age groups were described using 15-year intervals and using quartiles. Instances of nerve thickening were described for the left and right feet separately or, when describing correlation coefficients, as averages and as maxima for thickening in both feet. Bivariate correlations were described using Pearson correlation coefficients or Spearman correlation coefficients for skewed data distributions and their 95% confidence intervals. We used two-tailed analysis, and p-values were assumed significant when <0.05. Variables were shown stratified by sex and by age above the median of the study population. We used Chi square tests or Fisher exact tests depending on the expected count to describe differences of categorical data.

## Results

A total of 110 feet of 56 volunteers were examined within the time frame of one year. One foot was excluded due to a metatarsal bone fracture and another one due to gout. In all other volunteers, both feet were examined. One participant was excluded because of a potential history of fracture. No other participants were rejected.

A total of 30 volunteers (54.6%) were women and 26 (46.4%) were men. The median age was 28 and ranged from 20 to 69 years. Two participants (3.6 %) had a history of arthritis, and one (1.8%) presented with a previously diagnosed flexible flatfoot ( Table 1 ).

**Table:** **Table 1** Participant characteristics.

	Study participants (n = 56)
Age – median (range)	28 (20–69)
Female – n (%)	30 (53.6)
One-sided thickening – n (%)	15 (68.2)
Bilateral thickening – n (%)	7 (12.5)
Flatfoot – n (%)	1 (1.8)
Arthritis – n (%)	2 (3.6 )

Twenty-two individuals (39.3%) showed thickening of the MPPDN either in one or both feet. Specifically, 7 participants (12.5%) exhibited nerve thickening in both feet, 8 participants (14.3%) only in the right foot, and 7 (12.5%) only in the left foot. ( Table 2 ).

**Table:** **Table 2** Measurements of MPPDN thickenings. Abbreviations: CSA = cross-sectional area, MPPDN = medial plantar proper digital nerve.

	Left foot (14/54 feet – 25.9%)	Right foot (15/56 feet – 26.8%)	Total (22/56 participants – 39.3%)
CSA (mm ^2^ ) – median (range)	2.98 (1.44–4.68)	2.0 (0.74–4.30)	2.34 (1.44–4.68)
Length (mm) – median (range)	3.47 (2.21–6.47)	3.09 (1.55–4.77)	3.13 (2.21–6.47)

In 26 (89.7%) individuals, thickening was found at the level of the first interphalangeal (IP) joint making up the majority. Only two (6.9%) instances of thickening of the MDPPN were found proximal to the first IP joint and one (3.5%) was found distal to the first IP joint.

Out of the 110 examined feet, 29 (26.4%) showed a thickened MPPDN. In total, thickening was observed on the left side in 14/54 (25.9%) feet and on the right side in 15/56 (26.8%).

More participants above the median population age of 28 showed thickening of the MPPDN (15 [57.7] *versus* 7 [23.3], p = 0.009). The occurrence of nerve thickening was similar between women and men (10 [33.3] *versus* 12 [46.2], p = 0.327). The thickening of the MPPDN at the level of the interphalangeal joint showed a median CSA of 2.34 mm² (range: 1.44–4.68), with a slightly higher median on the left compared to the right side. The median thickening length was 3.13 mm (range: 2.21–6.47), again with the left side being slightly more pronounced ( Table 2 ).

Within our study sample, occurrences of MPPDN thickening increased across the age intervals of 15 years, 27.3% in the 15–29 years group, 41.7% in the 30–44 years group, 85% in the 45–59 years group, and 50% in the 60–74 years group ( Fig. 3 (a)). However, the number of participants varied across these groups, with the youngest group consisting of 33 participants and the oldest including only 4. These findings correspond to an increase from age quartiles one to four ( Fig. 3 (b)).

**Fig. 3:**
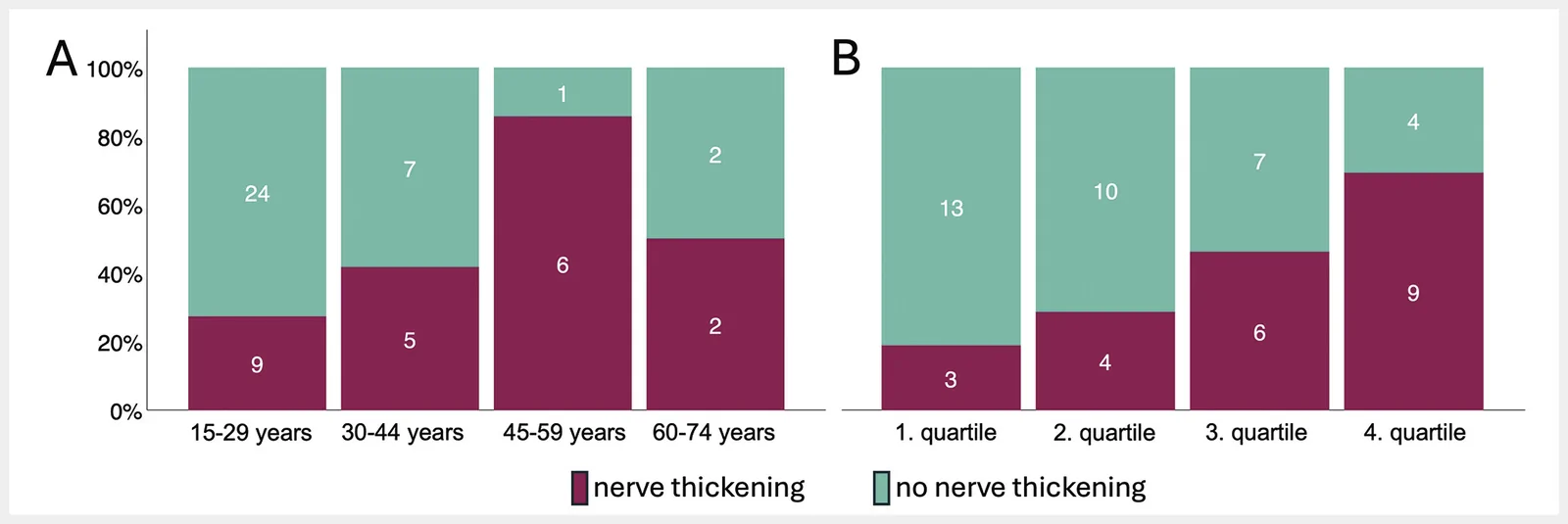
Occurrence of thickening of the medial plantar proper digital nerve (red) compared to no thickening (green). **A** : differences with increasing age of participants grouped into four age groups of 15 years, the number of participants varies in each group. **B** : grouped into age quartiles. All plots show the number of participants. Abbreviations: first quartile = age 20–24; second quartile = age 25–28; third quartile = age 29–40; fourth quartile = age 41–69.

CSA and length were plotted in Fig. 4 , CSA correlated with the length of the thickening of the MPPDN (Pearson r [95%CI], 0.714 [0.419–0.873], p < 0.01). Neither CSA nor length correlated with age (Spearman’s rho [95%CI], –0.132 [–0.534–0.318], p = 0.557 and 0.076 [0.737–0.369], p = 0.492) ( Fig. 5 ).

**Fig. 4:**
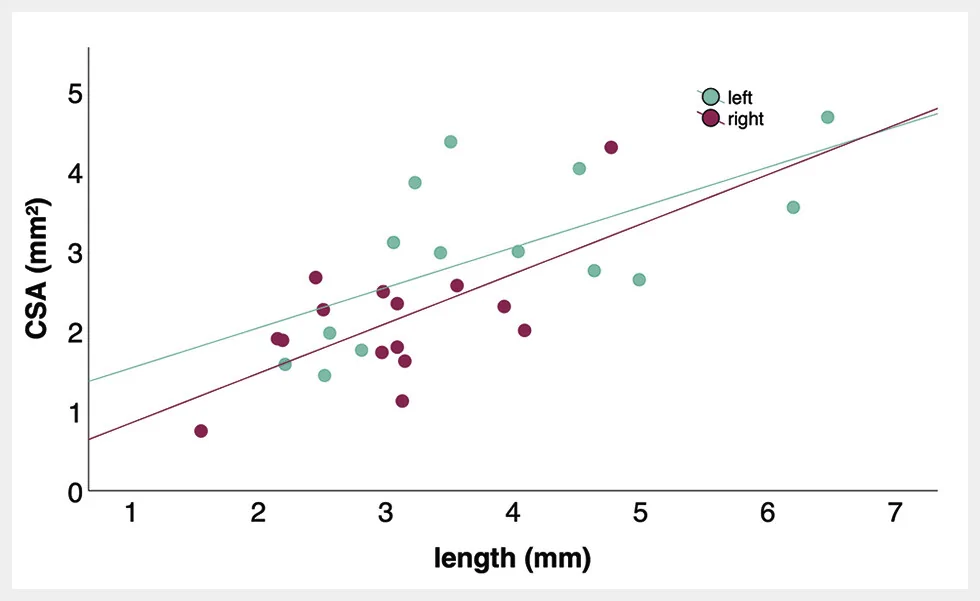
Correlation of cross-sectional area (CSA) and length of nerve.

**Fig. 5:**
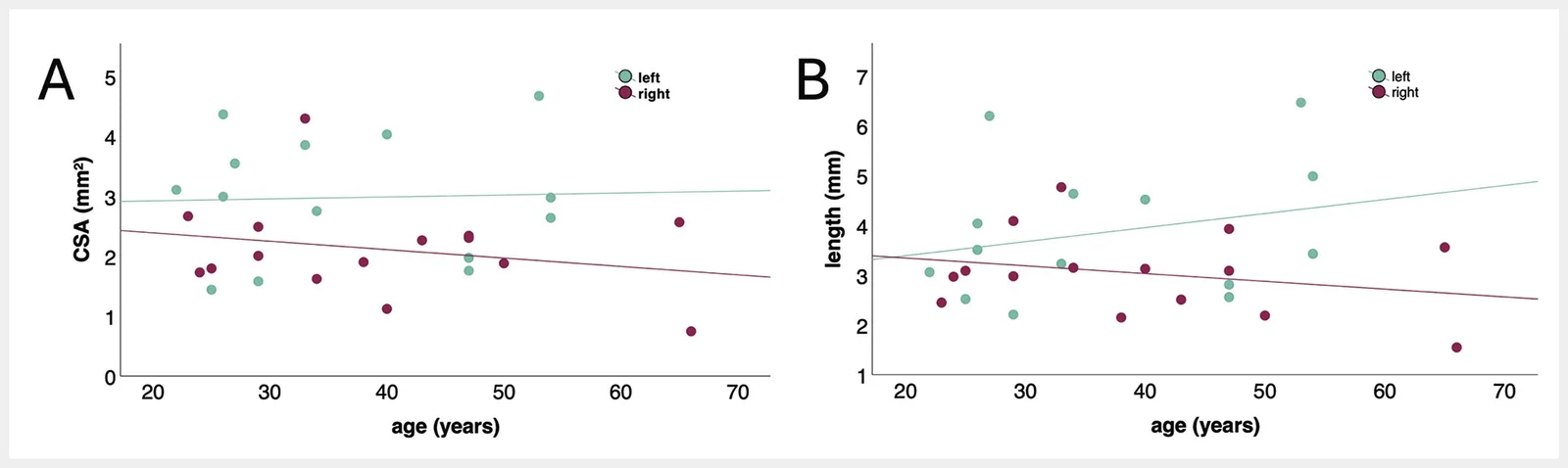
**A** : Correlation of cross-sectional area (CSA) and **B** : length of the nerve thickening to participant age.

## Discussion

This study found thickening of the MPPDN in 39.3% of participants and 26.4% of all feet, highlighting this phenomenon as a common occurrence. To the best of our knowledge, this study is the first to present imaging findings, anatomical location, and measurements of asymptomatic thickening of the MPPDN. We hypothesize that our findings may reduce the diagnostic value of ultrasound in the workup for Joplin’s neuroma.

Our primary finding was the detection of asymptomatic nerve thickening of the MPPDN. The number of cases was relatively large among all age groups, as the percentage of thickened nerves ranged from at least 27.3% to as high as 85%. We found only one study describing asymptomatic nerve enlargements, in which interdigital plantar nerves were examined, but not the MPPDN 12 . Nerve fibrosis and changes in nerve diameter may be the result of mechanical stress 13 14 . Consequently, changes in CSA are likely to develop at sites of compression or repeated mechanical irritation 15 16 . As suggested in the context of Mortonʼs neuroma 17 18 , we hypothesize that compression at the medial aspect of the big toe may frequently occur due to the wearing of tight-fitting shoes.

In previous reports, Joplin’s neuroma was described as focal MPPDN hypertrophy typical for a neuroma 1 2 . Considering the number of asymptomatic thickened nerves, it may be hypothesized that ultrasound imaging alone might lead to false-positive results regarding Joplin’s neuroma. Further studies, including symptomatic participants, are necessary to reproduce those findings and to determine if there is a CSA threshold or another determining ultrasound feature to differentiate between asymptomatic thickening and symptomatic Joplin’s neuroma. Thus, greater emphasis should be put on clinical examination to determine the diagnostic value of ultrasound findings. Moreover, differential diagnoses such as L5 radiculopathy or neuropathy of the superficial fibular nerve should be kept in mind for pain syndromes of the big toe.

This study was conducted at a single time point and exclusively on healthy individuals. Hence, no conclusions can be drawn as to whether these thickened nerves can be used as prognostic diagnostic markers. To establish these, follow-up studies as well as research including symptomatic populations are necessary.

In the analysis of nerve thickening in relation to participant characteristics, there is a higher occurrence of nerve thickening in the IP joint with increasing age, potentially indicating a link to nerve strain from repetitive microtrauma, which is also suspected as a possible cause of Joplinʼs neuroma 1 2 . Interestingly, this does not occur in the MCP joint, where one would suspect a higher mechanical stress due to the often protruding contour of the big toe. However, the majority of participants in this study were students, resulting in an underrepresentation of older participants. Further studies are needed to determine whether participants exhibiting thickening of the MPPDN show different loading patterns of the corresponding joint or engage in physical activities that place increased stress on the nerve, potentially contributing to the observed thickening.

To the best of our knowledge, our study is the first to compare CSA and the length of nerve thickening. We were able to show a correlation between CSA and the length of the increased nerve thickness, suggesting that nerve thickening can be quantified in both ways. We also investigated if the observed measurements were related to age, but we found a correlation of neither CSA nor length with participant age.

Our study is limited by sample size and age distribution. Especially percentages derived from subgroups could only be estimated with low precision due to the sample size. The cross-sectional design may introduce potential bias, limiting the ability to infer causality. Furthermore, the absence of symptomatic patients for comparison further restricts the robustness of the findings.

This study found a considerably high prevalence of asymptomatic thickening of the MPPDN in the IP joint in healthy volunteers of a broad range of ages. This finding highlights the importance of clinical examination and careful evaluation of ultrasound findings at this specific location. Further studies are needed to compare findings in asymptomatic and symptomatic individuals to determine if there are differentiating ultrasound features.
